# Molecular Dynamics Scoring of Protein–Peptide Models Derived from Coarse-Grained Docking

**DOI:** 10.3390/molecules26113293

**Published:** 2021-05-30

**Authors:** Mateusz Zalewski, Sebastian Kmiecik, Michał Koliński

**Affiliations:** 1 Biological and Chemical Research Center, Faculty of Chemistry, University of Warsaw, 1 Pasteura St., 02-093 Warsaw, Poland; mateusz.zalewski.fuw@gmail.com (M.Z.); sekmi@chem.uw.edu.pl (S.K.); 2 Bioinformatics Laboratory, Mossakowski Medical Research Centre, Polish Academy of Sciences, 5 Pawinskiego St., 02-106 Warsaw, Poland

**Keywords:** model scoring, protein–peptide docking, molecular dynamics, CABS-dock, coarse-grained docking

## Abstract

One of the major challenges in the computational prediction of protein–peptide complexes is the scoring of predicted models. Usually, it is very difficult to find the most accurate solutions out of the vast number of sometimes very different and potentially plausible predictions. In this work, we tested the protocol for Molecular Dynamics (MD)-based scoring of protein–peptide complex models obtained from coarse-grained (CG) docking simulations. In the first step of the scoring procedure, all models generated by CABS-dock were reconstructed starting from their original C-alpha trace representations to all-atom (AA) structures. The second step included geometry optimization of the reconstructed complexes followed by model scoring based on receptor–ligand interaction energy estimated from short MD simulations in explicit water. We used two well-known AA MD force fields, CHARMM and AMBER, and a CG MARTINI force field. Scoring results for 66 different protein–peptide complexes show that the proposed MD-based scoring approach can be used to identify protein–peptide models of high accuracy. The results also indicate that the scoring accuracy may be significantly affected by the quality of the reconstructed protein receptor structures.

## 1. Introduction

Protein–peptides interactions play essential roles in many cellular processes, and in recent years peptides have become attractive scaffolds for the design of new therapeutics [1]. Therefore, understanding the protein–peptide interactions and peptide-binding mechanisms is an important subject of theoretical and experimental research.

Computational prediction of protein–peptide complexes using molecular docking is a very challenging task [2]. First of all, peptides are very flexible molecules. Even relatively short peptides can adopt many distinct conformations and even marginal changes of their amino acid sequences may significantly alter their structure and dynamics. Additionally, even small changes in protein receptor three-dimensional structures can have qualitative effects on the binding mechanisms, binding energy, and structures of resulting complexes. For these reasons, molecular docking based on classical MD protocols is computationally extremely costly and, in most cases, not practical. Numerous approaches based on CG modeling strategies have been employed to deal with conformational sampling difficulties. For instance, the Rosetta FlexPepDock method [3] has proven quite effective in docking flexible peptides, providing the binding site can be approximately pre-assumed. On the contrary, another CG method, CABS-dock [4,5,6], enables simulations of global docking with the efficient exploration of docking poses over the entire receptor protein surface.

CABS-dock treats peptide ligands as fully flexible chains. The method allows for modeling structural changes of the receptor protein that are induced by peptide binding [6,7,8]. Appropriate coarse-graining of the protein/peptide structure and a very efficient Monte Carlo sampling of the system dynamics enable an exhaustive search of the conformational space and unrestrained docking of even quite long peptides (or small proteins). Several peptides can be docked simultaneously [9]. Of course, coarse-graining of conformational space and inevitable smoothening of energy surface (CABS uses knowledge-based statistical potentials—see the Materials and Methods section and References for more details) leads to imprecise energy scaling of the docked structures. Standard CABS-dock protocols generate a large number of protein–peptide structures, which are subject to CABS-energy ranking and structural clustering of the results. Usually, it is possible to identify a set of 1000 docking results containing a good resolution of the structure/s (close to the native structure) of the protein–peptide complex [4,5,6], although a selection of the single best structure is essentially impossible.

The problem of scoring protein–peptide poses is one of the major challenges in the field of molecular docking [2]. Scoring functions for protein–ligand docking can be divided into four categories [10]: physics-based using classical force field; empirical, with the binding affinity estimated by counting various protein–ligand interactions; knowledge-based, derived from statistics of close contacts observed in a representative set of protein–ligand structures; and machine-learning, where binding affinity is predicted with machine-learning algorithms (random forest, deep-learning, etc.). Since every class of scoring functions has its limitations, the most efficient are hybrid scoring methods, based on mixed functions [2,10]. The majority of protein–peptide docking tools—such as Rosetta FlexPepDock [3,11], HADDOCK [12], PyDockWEB [13], pepATTRACT [14], CABS-dock [4], and BiPPred [15]—use energy-based scoring that employs physics or knowledge-based terms, or their combination [2]. Some of those methods improve structure selection by predictions based on sequence similarities [15], template structure comparison [16], mutagenesis data [17], coevolution information [18], or structural clustering [4,6].

In this paper, we describe and test the MD-based strategy for scoring protein–peptide complex models provided by the CABS-dock method. The scoring is based on protein–peptide interaction energy values estimated from a series of short MD simulations for the sets of 1000 predicted models. Extensive MD simulations for this large number of evaluated models would be extremely computationally expensive, and at least for this reason not practical. Therefore, we propose a simpler, and as we show quite effective, protocol for ranking the large number of CG CABS-dock models using AA MD. First, we use the Modeller method [19] for reconstructions of atomistic structures of protein–peptide complexes. Next, these reconstructed structures are submerged in a water environment and subjected to short MD simulations with restrained C-alpha positions. These short simulations lead to a better arrangement of the amino acid side chains. Two AA force fields, CHARMM and AMBER, and the MARTINI CG force field are used. This way, the original CABS-dock models can be re-scored using calculated values of protein–peptide interaction energy. Finally, we analyze the quality of obtained best models in the sets of 10 and 100 top-scored models, respectively, and compare them with the original CABS-dock scoring results based on structural clustering. 

Additionally, we investigate the impact of the quality of reconstruction of protein receptor structure (mainly proper packing of protein side-chains) on the calculated protein–peptide interaction energy values and the resulting scoring accuracy. For this purpose, in all analyzed molecular models of protein–peptide complexes (reconstructed to AA representation), the structures of receptor proteins were substituted by their original AA crystal structures and the scoring procedure was repeated for all systems.

## 2. Results and Discussion

### 2.1. Comparison of MD-Based Scoring with CABS-Dock Scoring

An appropriate set of protein–peptide structures is needed to evaluate the performance of the scoring method. In particular, the benchmark set should contain a large population of alternative structural models of different protein–peptide complexes, with peptides that exhaustively sample the conformational space within the receptor binding site and its vicinity. For this purpose, we have chosen docking results for 66 unique protein–peptide complexes from the CABS-dock simulations [20]. The selected benchmark set includes high-resolution models for which calculated RMSD values for the C-alpha atoms of bound peptide ligands were below 4 Å when compared to the crystal structures. The different distribution profiles of RMSD values (see the first column in Supplementary Materials, Figure S1) indicate distinct ligand sampling patterns for the analyzed complexes, which should be taken into account when interpreting the scoring results.

In this work AA and CG MD simulations are used to score a large number of molecular models of protein–peptide complexes. All models were scored according to their energy of protein–peptide interactions estimated based on short MD simulations in explicit solvent. During the MD simulation, positional restraints were imposed on C-alpha atoms. This way, the geometries of amino acid side chains were optimized and the initial positions and conformations of the protein–peptide backbones were maintained during the MD simulations. The measured changes in RMSD values for C-alpha atoms of peptide ligands were small, not exceeding 1 Å for structures resulting from the AA MD and 1.8 Å for the CG MD simulations. To account for even small changes in peptide ligand conformation the RMSD values for the bound peptides were recalculated after each MD simulation. The observed relations between the measured RMSD values and interaction energy values for the group of 66 protein–peptide complexes are shown in Supplementary Materials, Figure S1.

The same MD-based scoring procedure was used to calculate the protein–peptide interaction energy for the crystal structures taken from the PDB database and used as reference structures for all scored complexes. Obtained energy values were marked on the plots with red dots (see Supplementary Information, Figure S1). Interestingly, only in 7 out of 66 analyzed crystal structures, the estimated protein–peptide interaction energy for crystal structures was lower than the interaction energy calculated for all the predicted models (PDB ID: 1iak, 1n7f, 1nln, 1ntv, 1ou8, 2hpl, and 3bwa). Additionally, for complexes having a significant population of models with bound peptide ligands located inside the receptor-binding site or in its vicinity (e.g., PDB ID: 1d4t, 1w93, 2dze, 3bfq), the interaction energies calculated for the crystal structures were lower than the interaction energies calculated for most of the predicted models.

**Figure 1:**
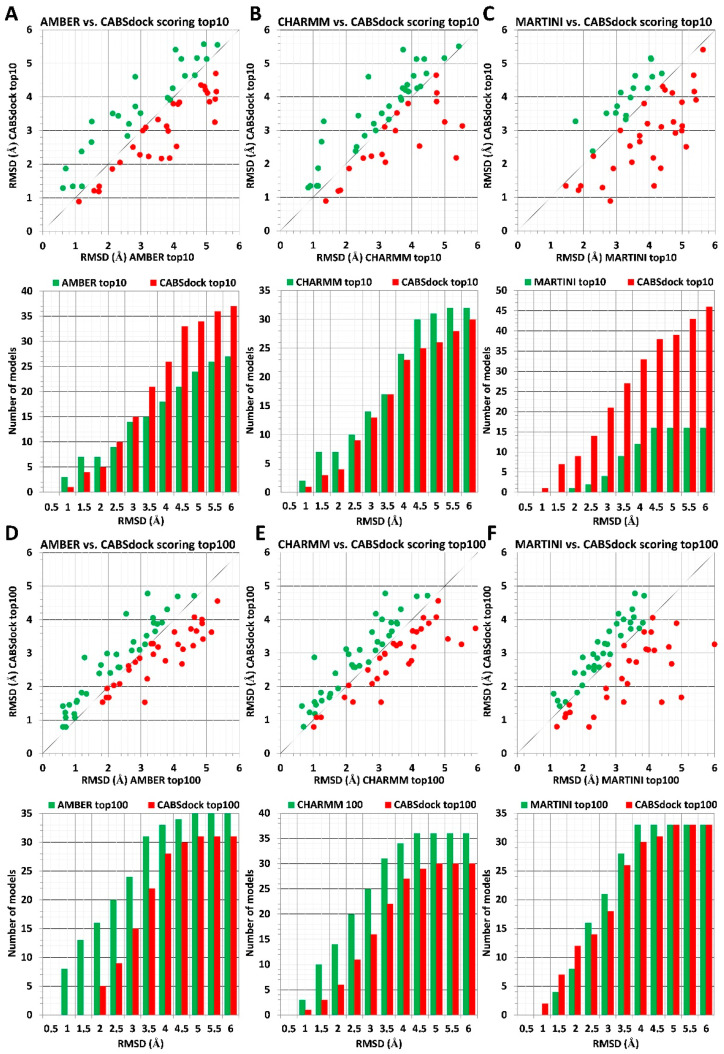
Comparison of MD-based versus CABS-dock scoring. The scoring data for 66 different protein–peptide complexes is shown. MD-based scoring was performed using three different force fields: AMBER, CHARMM, and MARTINI. Panels (**A**–**C**) show results obtained for sets of top 10 models, and panels (**D**–**F**) show results for sets of top 100 models. The plot at the top of each panel (**A**–**F**) shows RMSD values for best rank models according to the MD-based scoring method (color green) vs. RMSD values obtained for CABS-dock models (color red). At the bottom of each panel, the bar plot shows the number of models with the lower RMSD values identified using either the MD-based scoring method (color green) or using the CABS-dock scoring scheme (color red) for 0.5 Å intervals. Plots made based on data from Table S1.

The scoring results obtained using three different force fields (AMBER, CHARMM, and MARTINI) are compared to CABS-dock benchmark data in Figure 1. Upper panels (Figure 1A–C) show the analysis for 10 top-scored models, and lower panels (Figure 1D–F) show the analysis for 100 top-scored models. At the top of each panel, a plot shows RMSD values for the most accurate model out of 10 or 100 top-scored models for each of 66 protein–peptides complexes. The vertical y-axis corresponds to CABS-dock-derived RMSD values, whereas the horizontal x-axis corresponds to RMSD values calculated using the MD-based scoring procedure. Dots marked with the color green, which are positioned above the black diagonal line, indicate models characterized by lower RMSD values obtained using MD-based scoring. Similarly, red dots, positioned below the black diagonal line, indicate models with the lower RMSD values derived using the CABS-dock scoring approach. At the bottom of each panel, a bar plot shows the number of identified models with lower RMSD values using either the MD-based scoring procedure or the CABS-dock method calculated for 0.5 Å intervals.

Scoring results for the sets of 10 top-scored models indicate that the proposed MD-based scoring procedure using AA force fields of AMBER and CHARMM delivers more accurate scoring results than the default CABS-dock scoring. This is true for high-resolution complex structures with RMSD values below 2 Å (Figure 1A,B). Additionally, for identified models with resolution above 2 Å, scoring based on CHARMM parametrization performs slightly better than CABS-dock. In contrast, CABS-dock performs better for models above 2 Å when compared to MD-based scoring employing AMBER parametrization (Figure 1A,B).

When comparing the sets of 100 top-scored models, one can see the AA MD-based scoring approach’s superiority over the CABS-dock scoring. In the considered range of RMSD values from 0 to 6 Å, the AA MD-based procedure identified a significantly larger number of models with lower RMSD values when compared to CABS-dock results (Figure 1D,E).

In contrast to scoring results obtained using AA MD simulations, the CABS-dock scoring method shows much better performance when compared to scoring employing CG MARTINI force filed for sets of top 10 models, whereas both scoring methods showed similar accuracy for ranking sets of top 100 models (Figure 1C,F).

**Figure 2:**
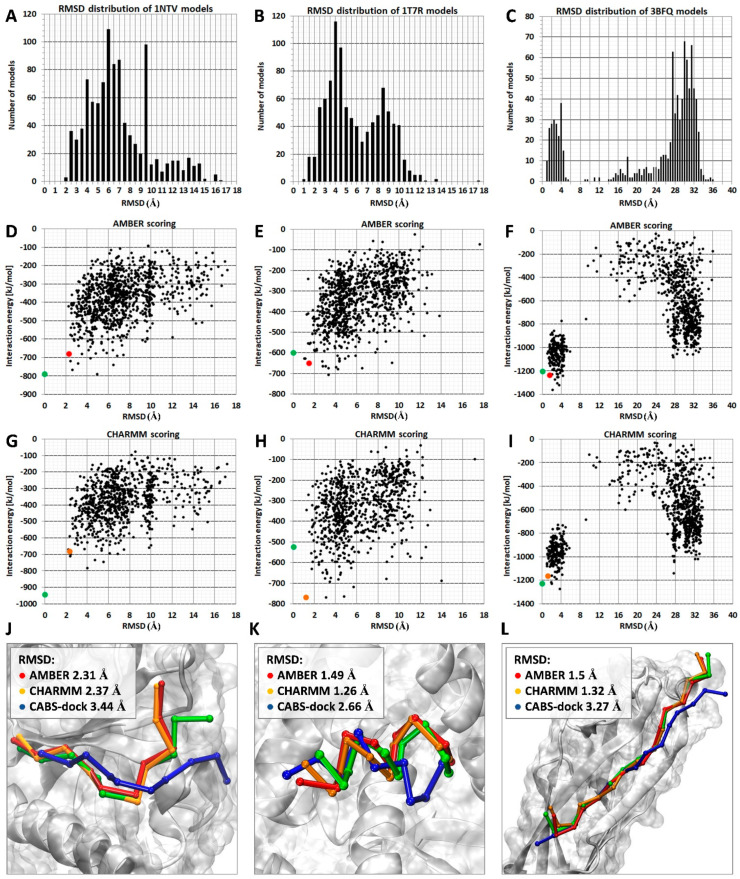
Examples of the MD-based scoring results. Ranking results for three complexes, 1ntv, 1t7r, and 3bfq, are shown. In panels (**A**–**C**) histograms of RMSD values calculated for model set derived from CABS-dock benchmark [20] are shown. Plots presenting RMSD values and corresponding interaction energy values calculated using AMBER and CHARMM force fields are shown in panels (**D**–**I**). Panels (**J**–**L**) show visualization of the best model found in the set of top 10 models for each of three complexes: CABS-dock model—color blue, the model scored using AMBER—color red, the model scored using CHARMM—color orange. For comparison, the crystal structure of peptide ligand is also displayed—color green.

An example of ranking results for the three protein–peptide complexes for which the AA MD-based scoring procedure provided more accurate results than the default CABS-dock scoring method is shown in Figure 2. Distribution plots of RMSD values calculated for C-alpha atoms of bound peptides are included in panels A, B, and C. As demonstrated, the sets of models for the first two systems (PDB IDs: 1ntv, 1t7r) have a similar distribution of RMSD values in the range from 2 to 12 Å, which means that the docked ligand extensively samples the receptor-binding site and its vicinity (Figure 2A,B). The set of models for the third complex (PDB ID: 3bfq) shows a bimodal RMSD distribution pattern with two noticeable peaks (Figure 2C). For the majority of the docked peptides, the calculated RMSD values fall within two separate bins, the first ranging from 1 to 4 Å and the second ranging from 28 to 32 Å. Those two clusters suggest that the peptide ligand can accept two distinct binding modes: one that closely resembles the reference structure; and another one located on the protein surface at a considerable distance from the receptor protein binding site.

Two plots showing RMSD values and corresponding interaction energy values calculated using AMBER and CHARMM parametrization for each scored set of models are presented in Figure 2D–I. The correlation between low values of interaction energy with low RMSD values calculated for bound peptides allows identification of the high accuracy models in the sets of top 10 models (red and orange dots).

The three peptide ligands in analyzed systems have similar peptide chain lengths (1ntv: 10 aa, 1t7r 10 aa, and 3bfq 15 aa), but when forming a complex, they adopt different secondary structures, including a coil-like conformation, a short α-helix, and an extended conformation forming a long β-sheet with parts of protein receptor. In Figure 2, in the bottom panels J, K, and L, top-scored models are compared with experimental structures. In the three presented systems, the proposed MD-based scoring procedure identified more accurate models than the CABS-dock scoring. The differences for the lowest RMSD values calculated for the set of top 10 models were above 1 Å in the case of 1ntv and 1t7r systems and about 2 Å in the 3bfq system.

### 2.2. Substitution of a Protein Receptor with Its Crystal Structure and Its Impact on Scoring Results

The CABS method uses discretized coordinates and a simplified representation of the protein chain. It allows very fast and efficient sampling of conformational space, which gives an advantage over other existing methods dedicated to protein–peptide docking. Docked ligands are fully flexible, and some structural changes are also allowed for receptor proteins that facilitate complex formation. However, this approach requires the reconstruction of full atomic details from C-alpha trace representation at the last stage of the docking procedure. This step is conducted using the Modeller method [19]. Unfortunately, the reconstruction process does not always assure the high quality of the local structure. This is due to inaccuracies in the C-alpha trace positions in the CG models and sometimes is not an optimal reconstruction of the backbone and side-chain positions (see our review [21]).

The molecular models of protein–peptide complexes used in this study were taken from the CABS-dock benchmark conducted for peptide-bound systems. It means that the initial structure of the receptor protein used for docking was taken directly from the protein–peptide crystallographic structure and presented bound conformation of the receptor protein. However, all resulting complex models contained a protein receptor whose structure was slightly different from its initial bound conformation due to the above-mentioned features of the CABS model, namely receptor flexibility during simulation followed by its reconstruction to AA representation. To analyze the effect of the changes in receptor structure (with respect to the input crystal structure) on the scoring results, we repeated the scoring procedure using AA MD with one modification—the receptor protein in scored complexes was replaced by the original structure taken from the crystallographic structure of the receptor–protein complex.

**Figure 3:**
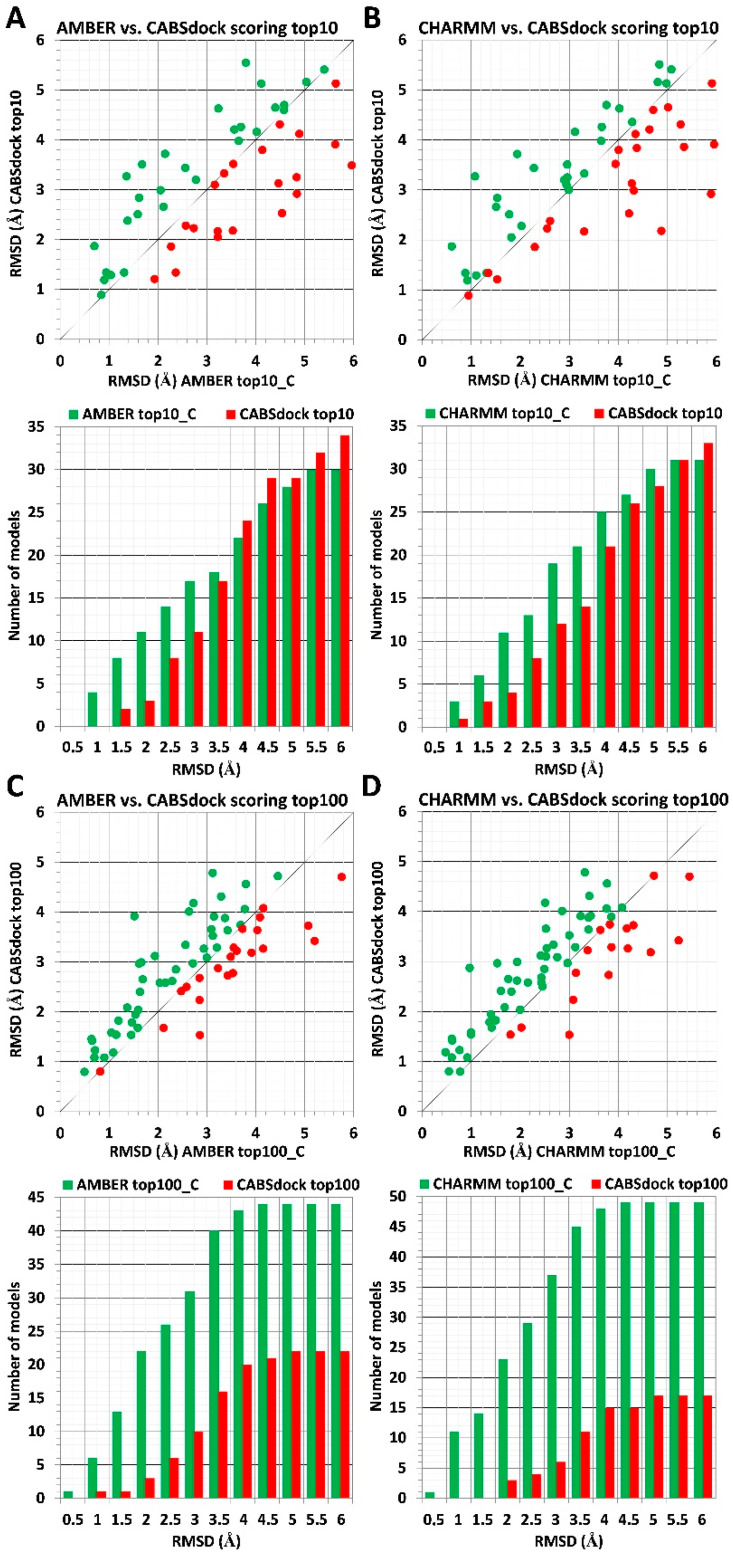
Comparison of MD-based versus CABS-dock scoring after substitution of a protein receptor with its crystal structure. The scoring data for 66 different protein–peptide complexes is shown. MD-based scoring was performed using two different force fields: AMBER and CHARMM. Interaction energy for each complex model was calculated using receptor–protein conformation derived from its crystal structure used in the CABS-dock benchmark (see Materials and Methods section for details). Panels (**A**,**B**) show results obtained for sets of top 10 models, and panels (**C**,**D**) show results for sets of top 100 models. The plot at the top of each panel (**A**–**D**) shows RMSD values for best rank models according to the MD scoring method (color green) vs. RMSD values obtained for CABS-dock models (color red). At the bottom of each panel, the bar plot shows numbers of models with lower RMSD values identified using either the MD scoring method (color green) or using the CABS-dock scoring scheme (color red) for 0.5 A intervals.

The ranking results indicate that the usage of receptor crystal structures significantly improves the scoring accuracy when compared to the CABS-dock driven data (see Figure 3). For scoring of sets of top 10 models, the MD-based procedure was able to find on average twice as many models presenting lower RMSD values in the RMSD range from 1 to 3 Å and in the range from 1 to 3.5 Å using AMBER and CHARMM parameters, respectively (Figure 3A,B). The data indicate that atomistic details of the receptor protein play a crucial role in estimating receptor–ligand interactions energy values, and even minor structural details may significantly impact the measurements. Ranking results for identified models with RMSD values above 4 Å show a similar performance to the AA MD-based and CABS-dock scoring approaches. Both methods were able to identify a similar number of best-scored models in analyzed RMSD intervals.

The AA MD-based scoring procedure showed a much better performance for scoring of sets of top 100 models. In the RMSD range from 1 to 4 Å, the significant majority of models presenting lower RMSD values were identified using the proposed scoring procedure. Besides, for less accurate complex structures presenting RMSD values above 4 Å, for about 2/3 of all analyzed cases, MD-based scoring was also able to deliver models with lower RMSD values.

The scoring results obtained using experimental receptor structures in the modeled complexes strongly suggest the need to improve all atom models’ reconstruction techniques and/or optimization strategies. In our experience, the improvement can be conducted primarily by enhancing the accuracy of backbone reconstruction since it significantly affects the subsequent side-chain reconstruction and energy-based scoring, as demonstrated in protein structure prediction [21,22]

## 3. Materials and Methods

### 3.1. The CABS-Dock Data Set

CABS-dock is an efficient method for global docking of peptides to known (or approximately known) receptor–protein structures. The CABS-dock is available as the webserver [4,5] or the standalone application [23]. In recent years, CABS-dock has been applied or extended to different modeling tasks. These include peptide docking with large structural changes of the receptor structure and disordered structures [7,8], docking with protein–peptide contact information [20], docking of peptides to GPCR structures [24] (see also recent review on CABS-dock development and applications [6]), and identification of peptide cleavage sites for protease–substrate systems [9].

For the docking simulations, CABS-dock uses the CABS CG model [25] based on an efficient Monte Carlo dynamics sampling scheme and knowledge-based statistical potentials describing interactions of amino acid united atoms (2–4 united atoms representing single residues, see review [26]). The water environment is treated in an implicit fashion where the solvent effects are encoded in burial and contact potentials describing interactions of united atoms. Such knowledge-based potentials work surprisingly well, although such a model of interactions is certainly imprecise and biased by “average” docking conditions. Consequently, while a subset of preferable docking results can be identified with an acceptable credibility, the best docking results are difficult to identify. Therefore, a better ranking of the docking results is highly desirable.

By default, CABS-dock generates 10,000 predictions in C-alpha trace representation of protein–peptide structures. The CABS-dock scoring procedure leads to the sets of 10 or 100 top-scored models and consists of two steps. First, 1000 top-scored models are selected based on CABS CG energy values (100 models having the lowest interaction energy are selected from every 10 replicas of a simulated system). Second, a set of 1000 top-scored models is subjected to structural clustering using a k-medoids algorithm, with the number k of clusters equal to 10 or 100, thus leading to 10 or 100 top-scored models.

In this work, we used the sets of models obtained from the CABS-dock docking protocol described by Blaszczyk et al. [20]. The protocol was validated on the PeptiDB benchmark set and the accuracy of individual docking cases was described in the Supplementary Information of the work [20]. Our working hypothesis is that MD-based scoring can distinguish high-accuracy models from medium-resolution models. To test this hypothesis, we selected the set of 66 different protein–peptide complexes presenting substantial content (at least a few or more) of high-accuracy models in the set of 1000 top-scored low energy models from the bound dataset. For each protein–peptide complex, the set of 1000 top-scored structures in C-alpha trace format was used as the input for the scoring procedure. For the list of all protein–peptide complexes (including PDB IDs) used in this study see Table S1.

### 3.2. Molecular Dynamics Scoring of Docking Results

**Figure 4:**
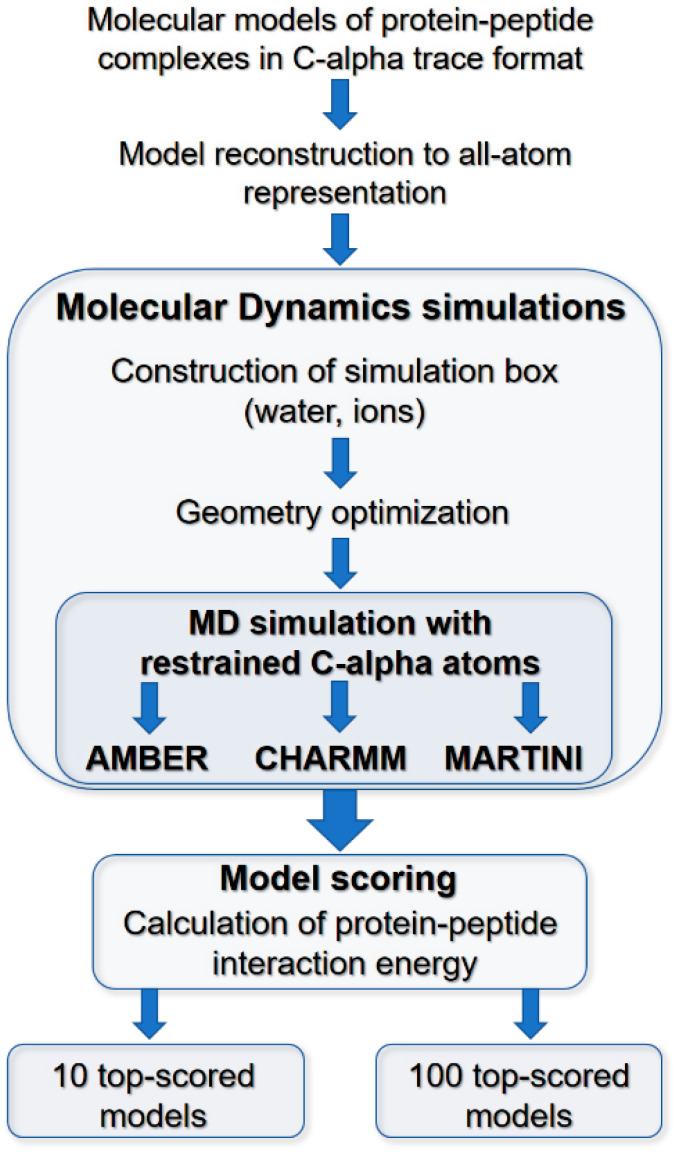
Main stages of the model scoring procedure. Scoring procedure includes: (i) reconstruction of C-trace models to AA representation; (ii) MD simulations of protein–peptide complexes in water environment using CHARMM, AMBER, and MARTINI parametrization; (iii) model scoring: calculation of protein–peptide interaction energy and RMSD values for bound peptide ligands.

The major stages of the scoring procedure are shown in Figure 4. The scoring has been conducted for 66 different protein–peptide complexes using the sets of 1000 molecular models predicted for each complex. First, complex structures in each set of models were reconstructed from C-alpha traces to their atomistic structures. The reconstruction was conducted using a Modeller-based protocol [19,27]. Next, for each analyzed complex, protein–peptide interaction energy was estimated for all models during short MD simulation using three different force-fields, two of the atomic resolution, CHARMM [28] and AMBER [29], and the CG MARTINI force-field [30]. Initially, each reconstructed AA structure of a complex was inserted in the cubic simulation box and solvated with water molecules. The simulation box dimensions were assigned for each system, considering the protein–peptide complex’s size to prevent interaction of simulated molecules with its periodic images. The specific number of ions was added to neutralize the system charge necessary for the particle mesh Ewald (PME) procedure [31] used to calculate electrostatic interactions.

CHARMM and AMBER-based scoring of AA models was conducted using the following procedure: First, the geometry of the system was optimized during 2000 steps of steepest descent algorithm. During the optimization, C-alpha atoms were kept at their initial positions to prevent distortions of the analyzed structure. In the next step, 100 ps MD simulations were conducted with position restraints using force constant 1000 kJ/(mol*nm^2^) imposed on C-alpha atoms of both the receptor protein and the peptide ligand. The simulation time step was 2 fs. Trajectory frames were recorded every 2 ps. The periodic boundary conditions were used. The temperature was set to 300 K and pressure was maintained at 1013 hPa. Long-range electrostatic interactions were approximated by the PME method [31] with a cutoff of 1.2 nm and hydrogen bonds were restrained using the LINCS algorithm [32]. In the last step, interaction energies were estimated for each of the last 25 frames of the recorded trajectory (which corresponds to the simulation period of 50 to 100 ps) as the sum of the energy of electrostatic and van der Waals interactions between protein and bound peptide. Average values of interaction energy were used to rank the analyzed model. The structure of the simulated complex derived from the last frame of the simulation trajectory was superimposed on its crystal structure (based on coordinates of receptor protein atoms only) and RMSD value was calculated for C-alpha atoms of the bound peptide.

A similar procedure for calculating the protein–peptide interaction energy described above was used for systems simulated in CG representation using the MARTINI model. Systems in atomistic coordinates were translated into CG representation and corresponding topologies were created using the martinize.py program. After optimization of the system geometry (1000 steps of the steepest descent algorithm), MD simulation was conducted with position restraints imposed on BB united atoms using force constant 1000 kJ/(mol*nm^2^). Due to the CG representation of the MARTINI model, a longer simulation step of 20 fs was used and the simulation for each analyzed model lasted 10 ns. A set of simulation parameters dedicated to the MARTINI force field was applied [33]. Trajectory frames were recorded every 20 ps. Simulations were conducted at temperature 310 K. Receptor–peptide interaction energy was calculated based on 250 trajectory frames recorded during the simulation time from 5 to 10 ns. The structure of the protein–peptide complex resulting from MD simulation was compared to the crystal structure, and RMSD value was calculated for BB united atoms of the bound peptide.

Calculated interaction energy and RMSD values for each model were used to rank sets of molecular models for 66 protein–peptide complexes. Results were presented for the best model in sets of top 100 models (model presenting the lowest RMSD value for peptide molecule out of 100 models scored with the lowest interaction energy value) and for the best model in the set of top 10 models (model presenting the lowest RMSD value for peptide molecule out of 10 models scored with the lowest interaction energy value). Finally, ranking results were compared to CABS-dock scoring (the lowest RMSD values for the sets of top 100 models and sets of top 10 models) based on structural clustering of the set of 1000 generated models for each protein–peptide complex (the CABS-dock data set). All simulations and data analysis were performed using the Gromacs program suit version 5.1.4 [34]. The VMD software version 1.9.4 was used for visualization of the obtained complex structures.

To investigate how the reconstruction quality of the receptor structure may impact the scoring results, we repeated the whole scoring procedure using AA force-fields for all 66 systems with one significant modification. In each scored complex model (resulting from CABS-dock docking simulation), the protein receptor structure was replaced by its crystal structure (derived from PDB database and also initially used as input for CABS-dock docking procedure). Scoring results were compared to previously collected data.

## 4. Conclusions

Efficient molecular docking of peptide ligands to protein receptors is a highly expected and necessary tool for a deeper understanding of many important molecular biology problems and, in particular, for the efficient design of new peptide-based drugs and therapies. Classical molecular docking tools have proven to be very efficient in studies of the interaction of small ligands with proteins and protein complexes, provided that the receptor structure is not significantly changed upon the docking and the binding site is approximately known. Peptides are specific ligands because they can adapt to a huge number of conformations and binding poses. Due to the large conformational space that needs to be explored, free docking of peptide ligands based on classical MD tools is now not possible. Only a significant simplification of the problem, based on CG modeling approaches, opens a possibility for attempting fully unrestrained docking of peptides. CABS-dock is such a tool that has proven to be quite effective in unrestrained and fully flexible docking of even quite large peptides (or small proteins) to protein receptors. CABS-dock docking generates huge numbers of possible structures for a relatively low computational cost, although selecting the best results is not easy at the CG modeling stage (due to the low resolution of the produced models and due to the smoothened energy surface). Therefore, a kind of multiscale ranking protocol is needed that merges CG with more precise AA modeling.

In summary, we proposed and tested a multiscale modeling protocol that allows more efficient use of the CABS-dock results for a very moderate cost of short MD simulations. The proposed re-ranking of the CABS-dock results works well for high and moderate resolution models; however, it is less efficient for the generally less accurate solutions. Analysis of the re-ranking results also indicated the necessity of very accurate AA reconstruction of the CG models. Additionally, it should be pointed out that the presented modeling protocol can be easily extended by unrestrained AA MD simulations aiming at long-timescale refinement of protein–peptide complexes. This is, however, much more expensive computationally and beyond the scope of the present work.

## Data Availability

The data presented in this study are available in supplementary material.

## Supplementary Materials

The following are available online, Figure S1: Graphical representation of scoring results for 66 protein–peptide complexes, Table S1: Model scoring results obtained for 66 protein–peptide complexes.

## Sample Availability

Samples of the compounds are not available from the authors.
