# Supplementary material for: Molecular Dynamics Scoring of Protein–Peptide Models Derived from Coarse-Grained Docking
Source: Molecules. 2021 May 30;26(11):3293. doi: 10.3390/molecules26113293 (PMC8197827; doi:10.3390/molecules26113293)
Supplement: Supplementary file 1 [file molecules-26-03293-s001.zip › molecules-1224710-supplementary.pdf]

Supplementary Materials

Molecular Dynamics scoring of protein-peptide molecular models derived from coarse-grained docking simulations

Mateusz Zalewski[1], Sebastian Kmiecik[1] and Michał Koliński[2*]

[1] *Biological and Chemical Research Center, Faculty of Chemistry, University of Warsaw, 1 Pasteura St., 02-093 Warsaw, Poland*
[2] *Bioinformatics Laboratory, Mossakowski Medical Research Centre, Polish Academy of Sciences, 5 Pawinskiego St., 02-106 Warsaw, Poland*

*Corresponding author: Michał Koliński, email: mkolinski@imdik.pan.pl

Table S1. Model scoring results obtained for 66 protein-peptide complexes. The lowest RMSD values are shown for sets of top100 and top10 models for CABS-dock scoring method and for MD-based scoring procedure using CHARMM, AMBER and MARTINI force fields. The scoring protocol employing AA simulations suing AMBER and CHARMM force field was repeated for all analyzed models with receptor protein substituted with its crystal structure.

**No.**	**PDB ID**	**Number of models**	**CABS-dock**		**CHARMM**				**AMBER**				**MARTINI**	
					**Receptor model**		**Receptor crystal**		**Receptor model**		**Receptor crystal**		**Receptor model**	
			**Top models:**											
			**100**	**10**	**100**	**10**	**100**	**10**	**100**	**10**	**100**	**10**	**100**	**10**
			**RMSD [A]**											
**1**	*1ce1*	1000	2.99	3.72	3.17	3.31	1.94	1.94	1.97	2.83	1.66	2.15	2.61	3.02
**2**	*1cka*	1000	3.65	4.16	3.39	3.90	2.52	3.12	3.43	5.30	3.09	4.02	2.80	5.37
**3**	*1czy*	1000	1.82	2.38	1.23	2.29	1.50	2.61	1.19	1.19	1.20	1.38	1.82	2.28
**4**	*1d4t*	1000	1.68	2.51	1.49	2.32	1.42	1.78	1.94	2.76	1.59	1.59	4.99	5.12
**5**	*1elw*	958	3.27	3.33	3.10	3.30	2.54	3.31	3.12	3.52	2.94	3.36	2.74	3.28
**6**	*1gyb*	1000	3.42	5.38	5.09	6.24	5.23	7.34	4.89	7.71	5.21	6.89	6.05	6.82
**7**	*1i8k*	931	2.58	2.53	2.27	4.23	2.43	4.22	2.35	4.09	2.04	4.54	2.49	3.41
**8**	*1iak*	1000	3.34	3.52	2.95	3.55	2.68	3.94	2.77	2.99	2.57	3.54	2.45	2.98
**9**	*1ihj*	1000	1.53	3.13	3.06	5.54	3.00	4.27	3.12	3.78	2.86	4.47	4.39	5.00
**10**	*1jd5*	808	3.29	3.49	3.64	7.16	3.86	6.04	3.31	6.09	3.21	5.96	6.51	11.95
**11**	*1jwg*	1000	2.85	4.21	3.02	3.80	2.49	4.63	2.96	4.97	2.37	3.56	2.43	4.48
**12**	*1kl3*	1000	4.72	5.16	4.47	4.99	4.73	4.80	4.62	4.71	4.45	5.04	3.86	4.04
**13**	*1klu*	1000	3.22	5.13	3.53	4.38	3.37	5.91	4.59	5.01	3.61	5.64	3.25	4.06
**14**	*1mvu*	1000	3.18	3.86	4.05	4.75	4.66	5.34	3.52	5.09	3.91	6.78	4.61	7.59
**15**	*1n7f*	1000	3.91	4.26	3.37	3.72	3.44	3.66	3.64	3.97	1.52	3.70	3.44	3.52
**16**	*1nln*	783	3.88	3.98	3.55	3.66	3.39	3.65	3.50	3.81	3.37	3.65	3.03	3.43
**17**	*1nq7*	761	1.08	1.87	1.10	1.15	0.60	0.60	0.70	0.70	0.70	0.70	2.32	4.35
**18**	*1ntv*	1000	2.58	3.44	2.23	2.37	2.16	2.28	2.31	2.31	2.15	2.56	2.17	3.29
**19**	*1nvr*	1000	2.87	3.84	1.03	7.08	0.97	4.38	1.28	4.17	3.23	6.95	1.99	4.99
**20**	*1oai*	898	3.74	6.25	5.93	6.52	3.83	3.83	6.03	6.67	3.69	4.32	3.71	6.46
**21**	*1ou8*	1000	3.64	5.13	4.14	4.14	3.39	4.98	4.02	4.23	4.03	4.12	3.86	6.31
**22**	*1se0*	1000	3.73	5.57	4.28	6.95	4.31	6.64	4.52	4.92	5.08	6.71	3.45	6.90
**23**	*1svz*	621	2.04	2.23	2.08	2.77	2.00	2.55	2.17	3.23	1.60	2.73	1.98	2.30
**24**	*1t4f*	1000	2.50	3.2	2.66	2.84	2.45	2.89	2.63	2.63	2.59	2.78	2.33	3.94
**25**	*1t7r*	1000	1.58	2.66	1.26	1.26	1.00	1.51	1.05	1.49	1.05	2.11	1.22	3.71
**26**	*1tp5*	974	1.19	1.21	1.05	1.83	0.47	1.53	0.96	1.57	1.09	1.93	1.48	1.84
**27**	*1tw6*	717	2.65	2.99	2.18	3.50	1.76	4.31	1.78	3.83	1.69	2.05	2.77	4.99
**28**	*1uj0*	1000	3.63	2.92	2.79	10.10	3.63	5.89	5.15	10.00	3.42	4.84	4.07	4.80
**29**	*1w9e*	1000	0.80	1.29	0.70	0.85	0.54	1.10	0.62	0.62	0.82	1.04	1.20	2.58
**30**	*1x2r*	820	3.91	3.91	3.54	3.69	3.23	5.95	3.39	3.89	3.15	5.63	3.82	5.42
**31**	*1ymt*	889	3.08	3.1	2.89	3.18	2.75	2.94	2.72	3.15	3.00	3.16	4.17	4.37
**32**	*1yph*	900	4.08	4.65	4.74	4.74	4.07	5.01	4.63	4.65	4.16	4.40	3.54	5.35
**33**	*1yuc*	1000	1.68	2.28	1.95	3.10	2.02	2.02	2.02	2.97	2.12	2.57	2.72	6.43
**34**	*1ywo*	1000	3.89	5.01	4.51	6.47	3.86	6.30	4.87	8.19	4.09	6.48	4.84	7.38
**35**	*1zuk*	1000	3.52	3.8	3.31	3.89	3.00	4.01	3.17	3.99	3.12	4.14	3.15	3.84
**36**	*2ak5*	1000	2.77	4.31	3.98	4.27	3.13	5.27	3.75	4.94	3.54	4.49	3.40	4.41
**37**	*2b9h*	1000	2.97	4.13	3.18	10.51	2.96	10.63	3.38	10.33	2.72	10.69	2.31	3.13
**38**	*2bba*	1000	3.66	3.94	4.02	6.48	4.17	6.75	4.69	5.27	3.73	9.54	9.26	9.55
**39**	*2c3i*	836	3.10	4.36	2.41	3.86	2.52	4.28	2.96	4.83	3.49	6.39	3.98	6.90
**40**	*2cch*	957	4.56	5.55	4.80	6.41	3.77	6.53	5.33	5.33	3.80	3.80	6.59	8.32
**41**	*2d0n*	1000	4.06	4.7	4.35	4.44	3.76	3.76	3.36	5.29	3.78	4.58	4.12	4.38
**42**	*2ds8*	1000	1.08	1.19	1.24	1.76	0.92	0.92	0.98	1.72	0.90	0.90	1.45	6.43
**43**	*2dze*	1000	1.42	1.34	0.65	0.91	0.61	0.88	0.61	1.20	0.66	1.31	1.29	1.46
**44**	*2fnt*	857	1.23	1.34	0.89	1.15	0.76	1.31	0.69	0.92	0.71	0.94	1.60	1.92
**45**	*2foj*	724	4.01	5.51	3.09	5.43	2.85	4.83	4.86	9.76	2.64	8.69	3.23	10.29
**46**	*2fvj*	748	1.46	1.34	1.08	1.11	0.60	1.35	0.81	1.73	0.64	2.37	1.58	4.15
**47**	*2h9m*	1000	1.79	1.86	1.52	2.10	1.37	2.30	1.33	2.12	1.47	2.27	1.11	2.90
**48**	*2hpl*	1000	2.41	4.63	3.21	4.06	1.61	4.02	2.06	4.34	2.48	3.24	1.75	3.57
**49**	*2iv9*	1000	2.73	4.6	2.68	2.68	3.80	4.71	2.83	2.83	3.43	4.58	3.62	4.09
**50**	*2j6f*	760	2.68	6.99	3.91	12.73	2.43	12.52	4.25	5.50	2.85	12.60	4.69	11.87
**51**	*2jam*	1000	4.18	6.12	2.90	8.73	2.51	2.51	2.55	3.42	2.73	2.87	3.08	9.21
**52**	*2o02*	885	4.78	5.41	3.19	3.73	3.31	5.08	3.20	4.05	3.12	5.40	3.58	5.63
**53**	*2o4j*	449	1.54	2.17	2.21	2.52	1.80	3.30	1.83	3.62	1.45	3.22	3.24	15.40
**54**	*2otu*	1000	4.70	6.74	4.15	6.74	5.45	6.36	4.12	6.50	5.76	12.78	6.30	7.52
**55**	*2p1k*	1000	2.62	3	2.40	2.90	1.93	2.99	2.62	3.06	2.29	7.24	2.34	3.12
**56**	*2p1t*	1000	0.79	0.89	1.01	1.39	0.77	0.95	0.71	1.11	0.50	0.84	2.18	2.81
**57**	*2p54*	1000	2.23	2.18	2.94	5.35	3.08	4.88	3.19	3.87	2.85	3.53	3.18	4.12
**58**	*2puy*	1000	1.94	3.51	1.75	3.09	1.40	2.96	1.97	2.14	1.54	1.68	2.70	2.70
**59**	*2pv2*	1000	2.40	2.05	1.67	3.20	1.82	1.82	1.72	2.36	1.64	3.22	2.01	3.47
**60**	*2qos*	973	3.29	4.12	3.44	4.76	3.12	4.35	3.37	5.03	3.55	4.89	2.66	4.70
**61**	*2r7g*	944	3.12	2.99	2.00	10.69	2.42	8.41	4.28	10.66	1.94	10.72	3.92	9.66
**62**	*2vj0*	1000	3.26	3.79	5.51	7.24	4.19	6.27	4.13	4.13	4.15	6.15	6.00	7.55
**63**	*2zjd*	1000	2.09	3.25	2.79	5.00	1.68	2.96	2.35	5.25	1.37	4.84	3.35	4.73
**64**	*3bfq*	1000	1.54	3.27	1.04	1.32	1.00	1.08	1.03	1.50	1.15	1.36	1.47	1.76
**65**	*3bwa*	941	2.97	2.84	2.07	2.57	1.53	1.53	2.25	2.59	1.62	1.62	2.82	3.71
**66**	*3cvp*	1000	4.31	4.26	3.66	4.16	3.40	6.15	3.89	7.02	3.29	6.36	3.49	3.95

Figure S1. Graphical representation of scoring results for 66 protein-peptide complexes. In each row plots presenting data corresponding to one scored complex are shown. At the left side PDB ID of each complex is shown with the number of residues of docked peptide ligand. Plots form left to right show as follows: distribution of RMSD values calculated for peptide ligand in scored set of models; RMSD values calculated for docked ligand vs. protein-peptide interaction energies estimated using CHARMM, AMBER and MARTINI force fields. Red dotes indicate RMSD/interaction energy values calculated using crystal structures.

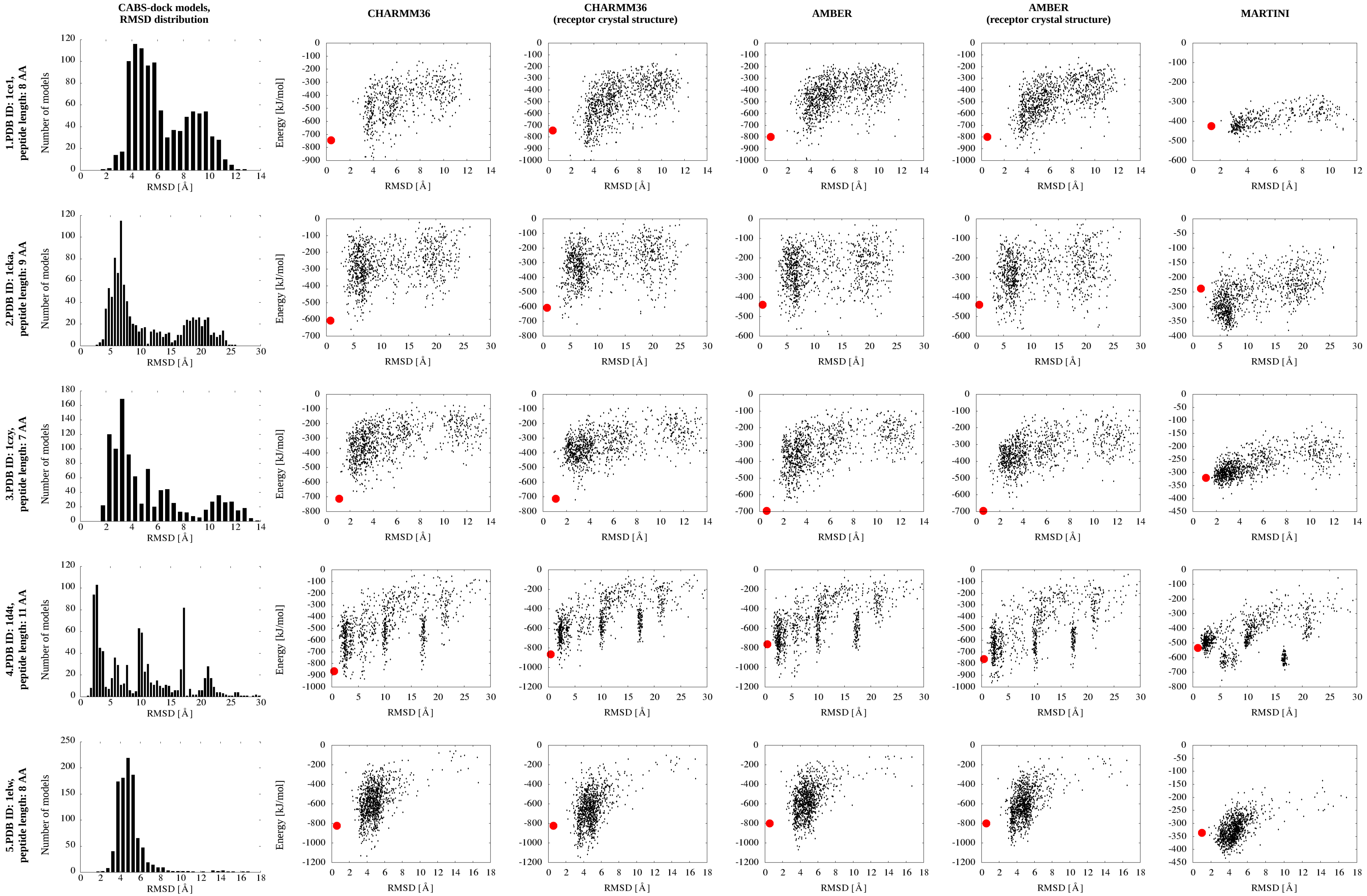

CABS-dock models, RMSD distribution
CHARMM36
CHARMM36 (receptor crystal structure)
AMBER
AMBER (receptor crystal structure)
MARTINI
1.PDB ID: 1ce1, peptide length: 8 AA
2.PDB ID: 1cka, peptide length: 9 AA
3.PDB ID: 1czy, peptide length: 7 AA
4.PDB ID: 1d4t, peptide length: 11 AA
5.PDB ID: 1elw, peptide length: 8 AA
Number of models
Energy [kJ/mol]
RMSD [Å]

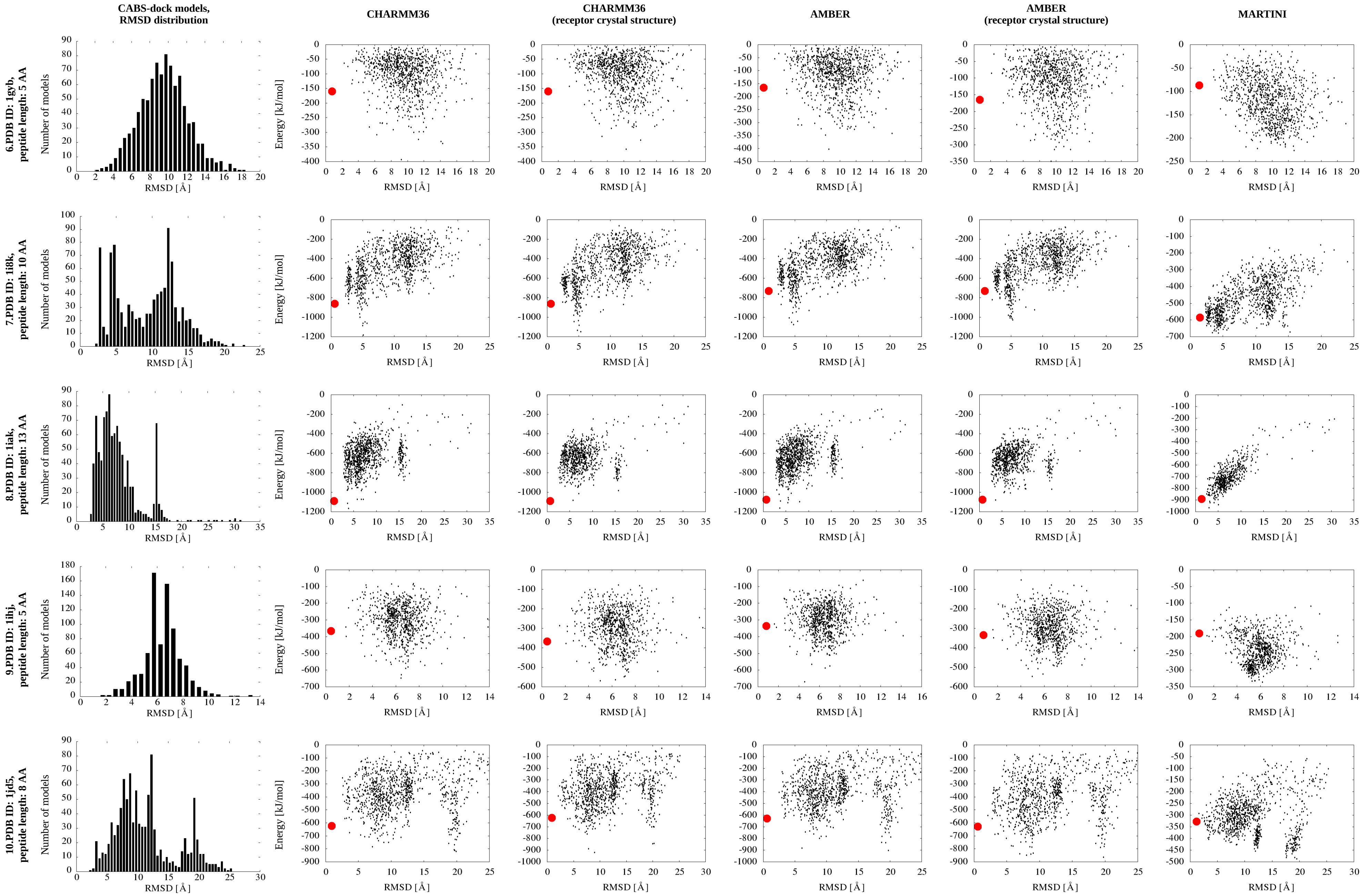
CABS-dock models,
RMSD distribution
CHARMM36
CHARMM36
(receptor crystal structure)
AMBER
AMBER
(receptor crystal structure)
MARTINI
6.PDB ID: 1gyb,
peptide length: 5 AA
7.PDB ID: 1i8k,
peptide length: 10 AA
8.PDB ID: 1iak,
peptide length: 13 AA
9.PDB ID: 1lhj,
peptide length: 5 AA
10.PDB ID: 1jd5,
peptide length: 8 AA
Number of models
Energy [kJ/mol]
RMSD [Å]

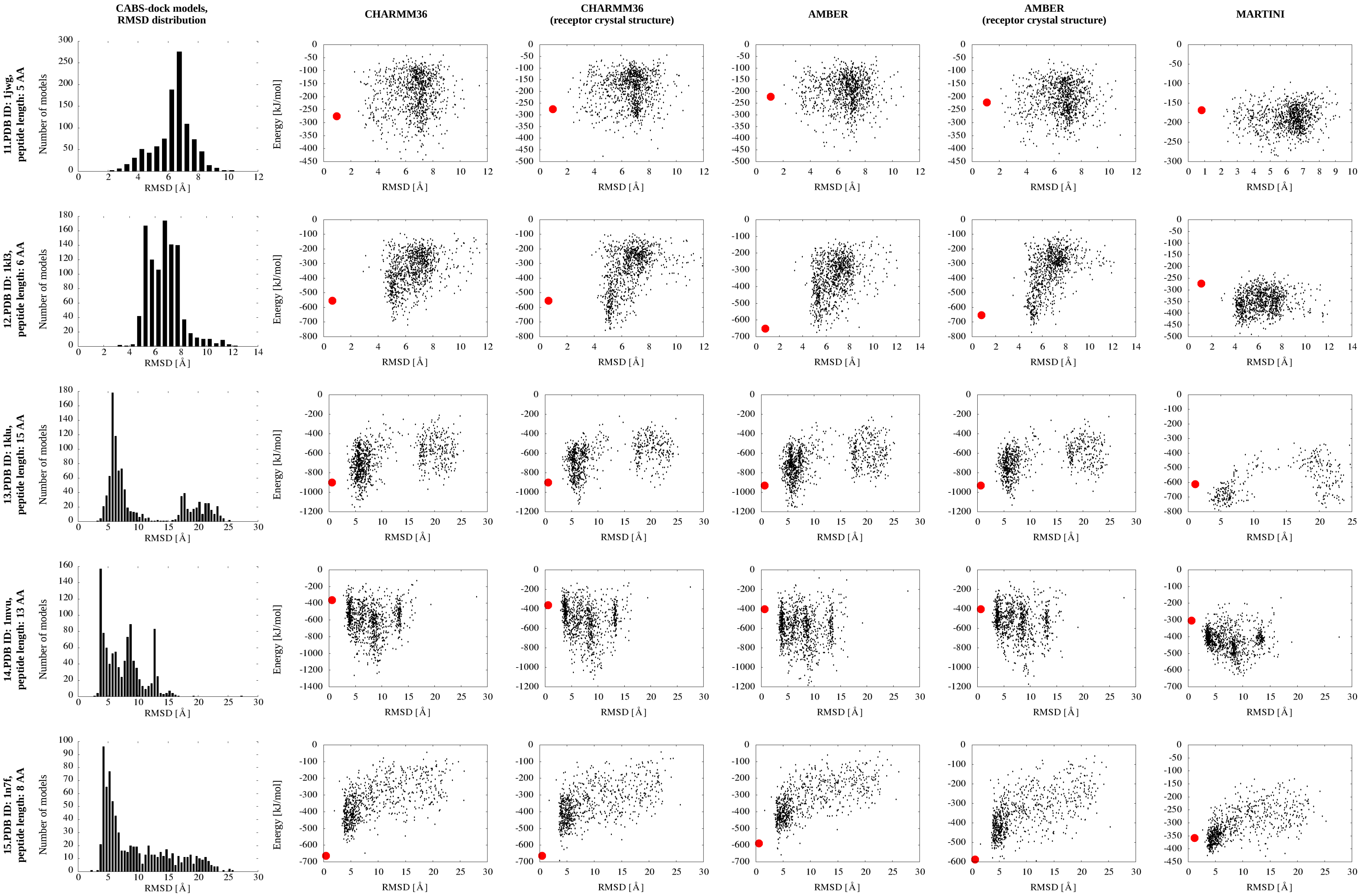

CABS-dock models, RMSD distribution
CHARMM36
CHARMM36 (receptor crystal structure)
AMBER
AMBER (receptor crystal structure)
MARTINI
11.PDB ID: 1jwg, peptide length: 5 AA
12.PDB ID: 1kl3, peptide length: 6 AA
13.PDB ID: 1klu, peptide length: 15 AA
14.PDB ID: 1mvu, peptide length: 13 AA
15.PDB ID: 1n7f, peptide length: 8 AA
Number of models
Energy [kJ/mol]
RMSD [Å]

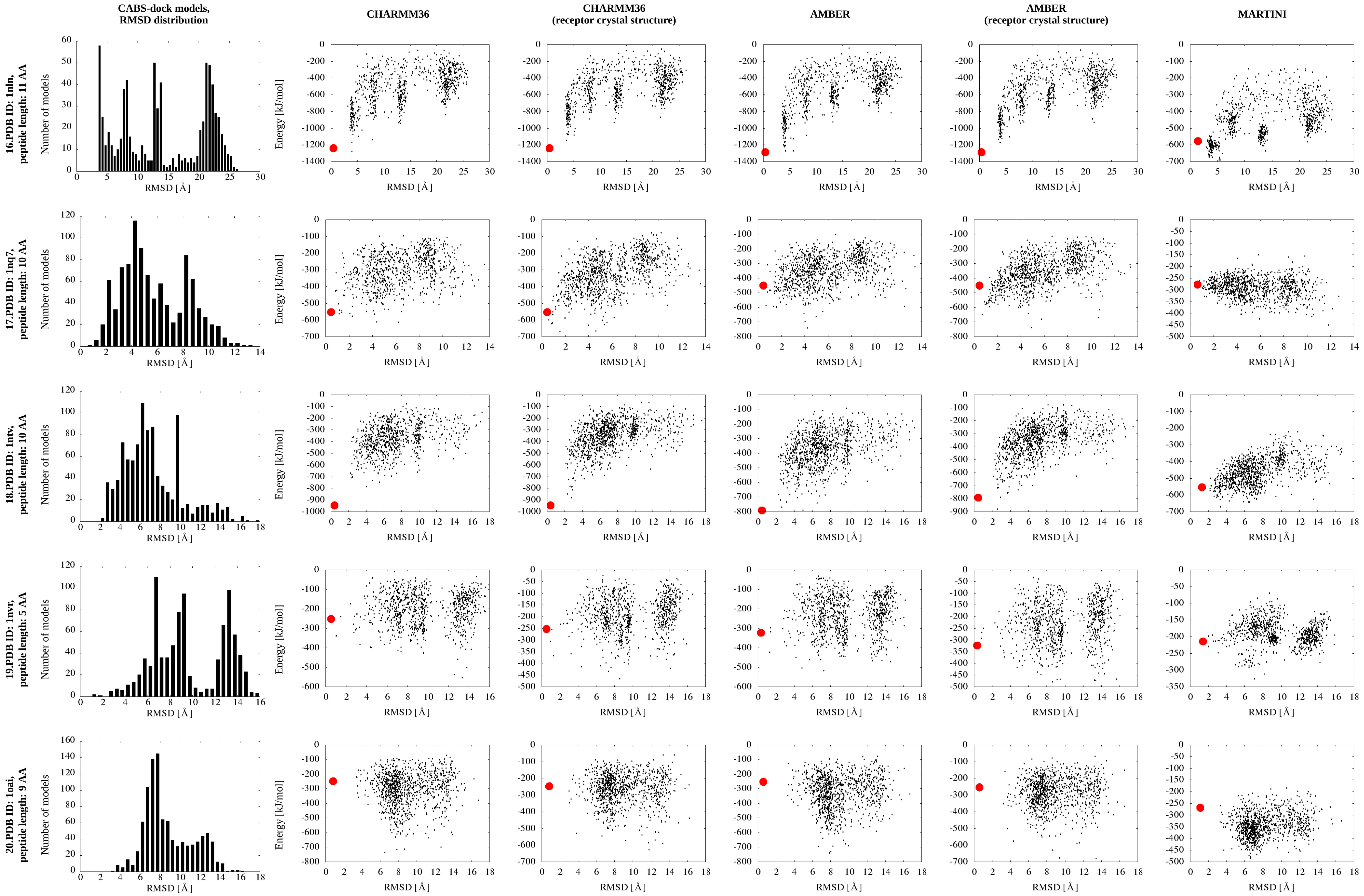

CABS-dock models, RMSD distribution
CHARMM36
CHARMM36 (receptor crystal structure)
AMBER
AMBER (receptor crystal structure)
MARTINI
16.PDB ID: 1nln, peptide length: 11 AA
17.PDB ID: 1nq7, peptide length: 10 AA
18.PDB ID: 1ntv, peptide length: 10 AA
19.PDB ID: 1nvr, peptide length: 5 AA
20.PDB ID: 1oai, peptide length: 9 AA
Number of models
Energy [kJ/mol]
RMSD [Å]

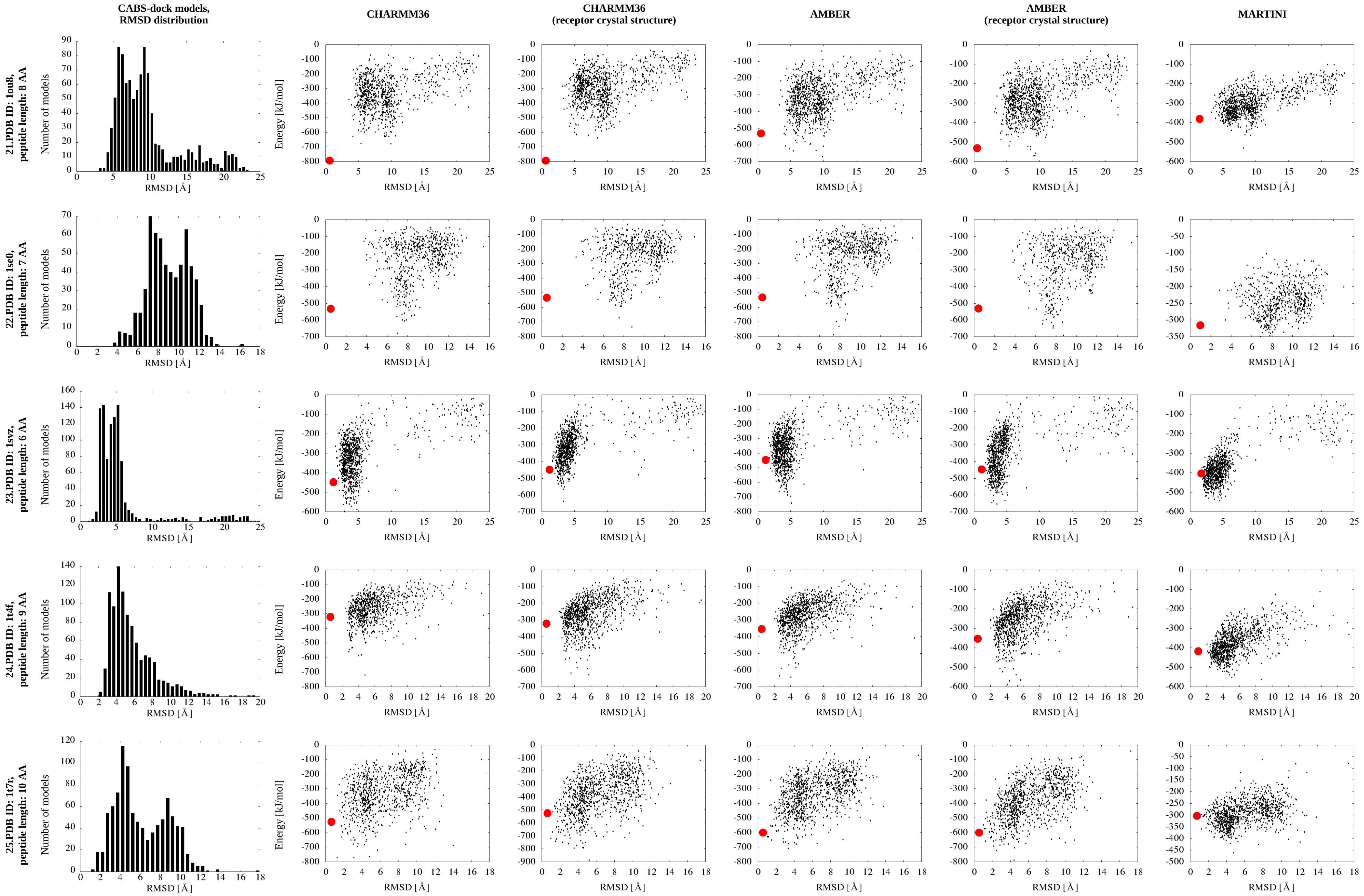

CABS-dock models,
RMSD distribution
CHARMM36
CHARMM36
(receptor crystal structure)
AMBER
AMBER
(receptor crystal structure)
MARTINI
21.PDB ID: 1ou8,
peptide length: 8 AA
22.PDB ID: 1se0,
peptide length: 7 AA
23.PDB ID: 1svz,
peptide length: 6 AA
24.PDB ID: 1t4f,
peptide length: 9 AA
25.PDB ID: 1t7r,
peptide length: 10 AA
Number of models
Energy [kJ/mol]
RMSD [Å]

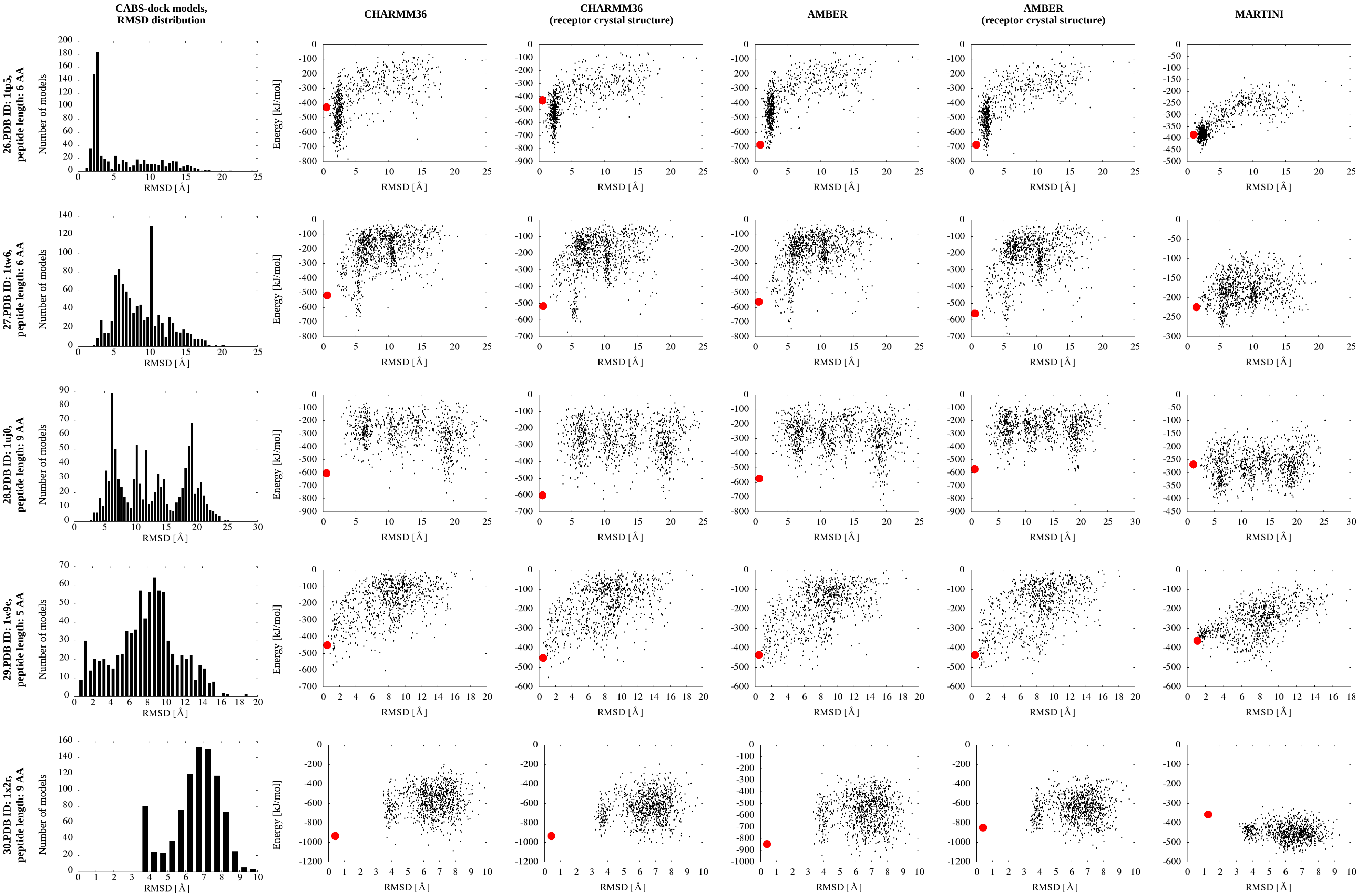
CABS-dock models,
RMSD distribution
CHARMM36
CHARMM36
(receptor crystal structure)
AMBER
AMBER
(receptor crystal structure)
MARTINI
26.PDB ID: 1tp5,
peptide length: 6 AA
27.PDB ID: 1tw6,
peptide length: 6 AA
28.PDB ID: 1uj0,
peptide length: 9 AA
29.PDB ID: 1w9e,
peptide length: 5 AA
30.PDB ID: 1x2r,
peptide length: 9 AA
Number of models
Energy [kJ/mol]
RMSD [Å]

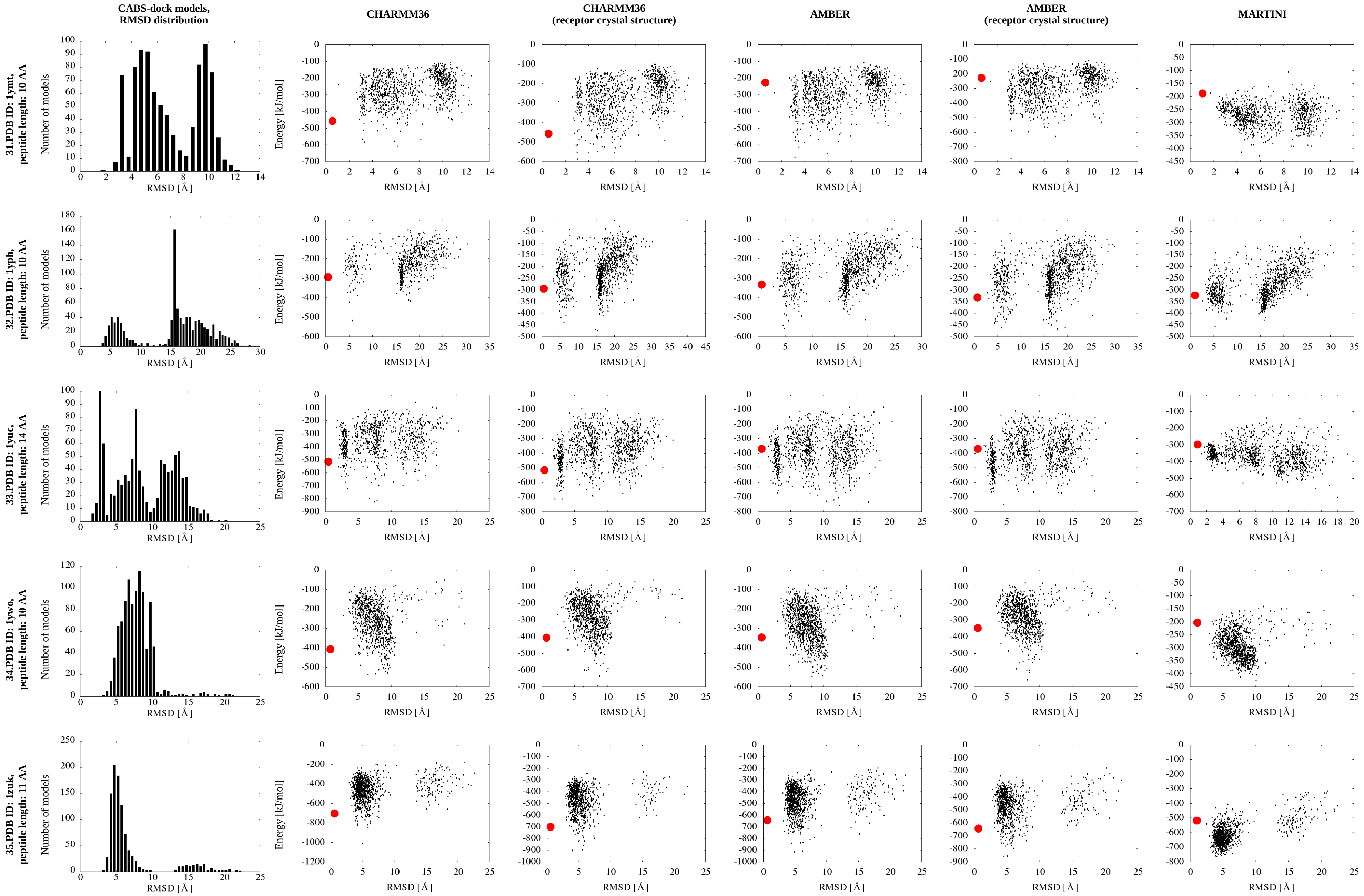

CABS-dock models,
RMSD distribution
CHARMM36
CHARMM36
(receptor crystal structure)
AMBER
AMBER
(receptor crystal structure)
MARTINI
31.PDB ID: 1ymt,
peptide length: 10 AA
32.PDB ID: 1yph,
peptide length: 10 AA
33.PDB ID: 1yuc,
peptide length: 14 AA
34.PDB ID: 1ywo,
peptide length: 10 AA
35.PDB ID: 1zuk,
peptide length: 11 AA
Number of models
Energy [kJ/mol]
RMSD [Å]

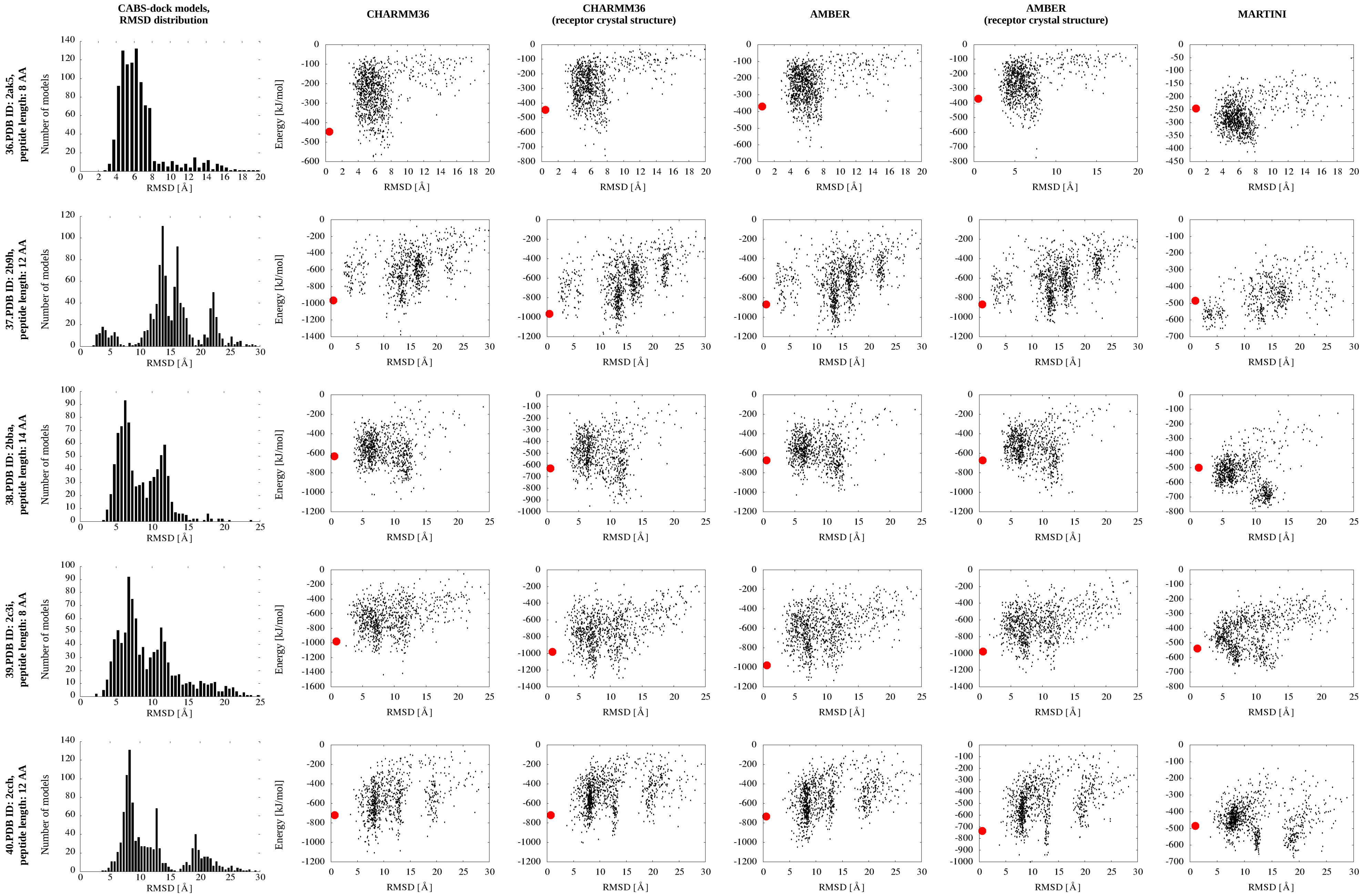

CABS-dock models,
RMSD distribution
CHARMM36
CHARMM36
(receptor crystal structure)
AMBER
AMBER
(receptor crystal structure)
MARTINI
36.PDB ID: 2ak5,
peptide length: 8 AA
37.PDB ID: 2b9h,
peptide length: 12 AA
38.PDB ID: 2bba,
peptide length: 14 AA
39.PDB ID: 2c3i,
peptide length: 8 AA
40.PDB ID: 2cch,
peptide length: 12 AA
Number of models
Energy [kJ/mol]
RMSD [Å]

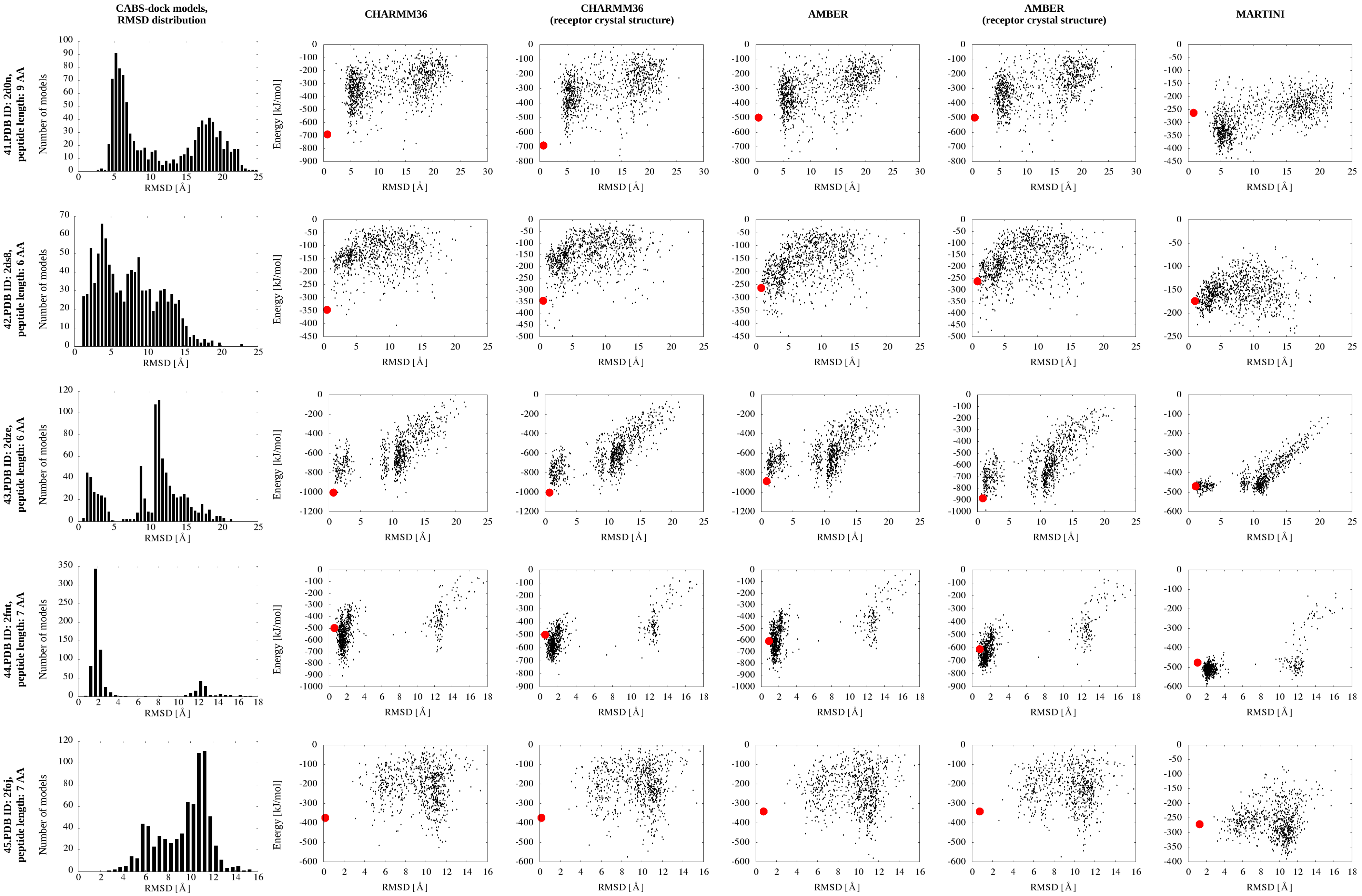

CABS-dock models, RMSD distribution
CHARMM36
CHARMM36 (receptor crystal structure)
AMBER
AMBER (receptor crystal structure)
MARTINI
41.PDB ID: 2d0n, peptide length: 9 AA
42.PDB ID: 2ds8, peptide length: 6 AA
43.PDB ID: 2dze, peptide length: 6 AA
44.PDB ID: 2fmt, peptide length: 7 AA
45.PDB ID: 2foj, peptide length: 7 AA
Number of models
Energy [kJ/mol]
RMSD [Å]

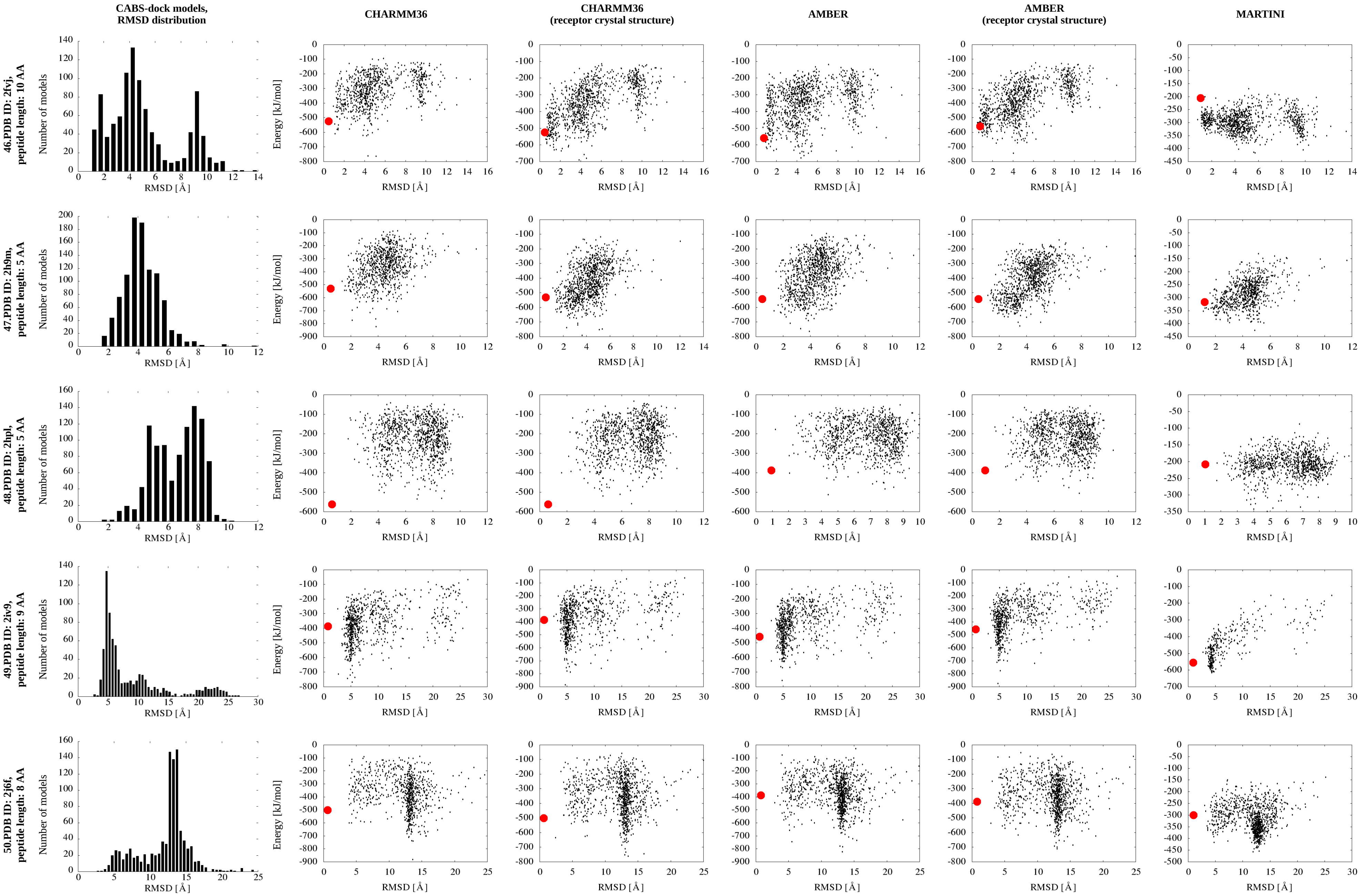
CABS-dock models, RMSD distribution
CHARMM36
CHARMM36 (receptor crystal structure)
AMBER
AMBER (receptor crystal structure)
MARTINI
46.PDB ID: 2fvj, peptide length: 10 AA
47.PDB ID: 2h9m, peptide length: 5 AA
48.PDB ID: 2hpl, peptide length: 5 AA
49.PDB ID: 2iv9, peptide length: 9 AA
50.PDB ID: 2j6f, peptide length: 8 AA
Number of models
Energy [kJ/mol]
RMSD [Å]

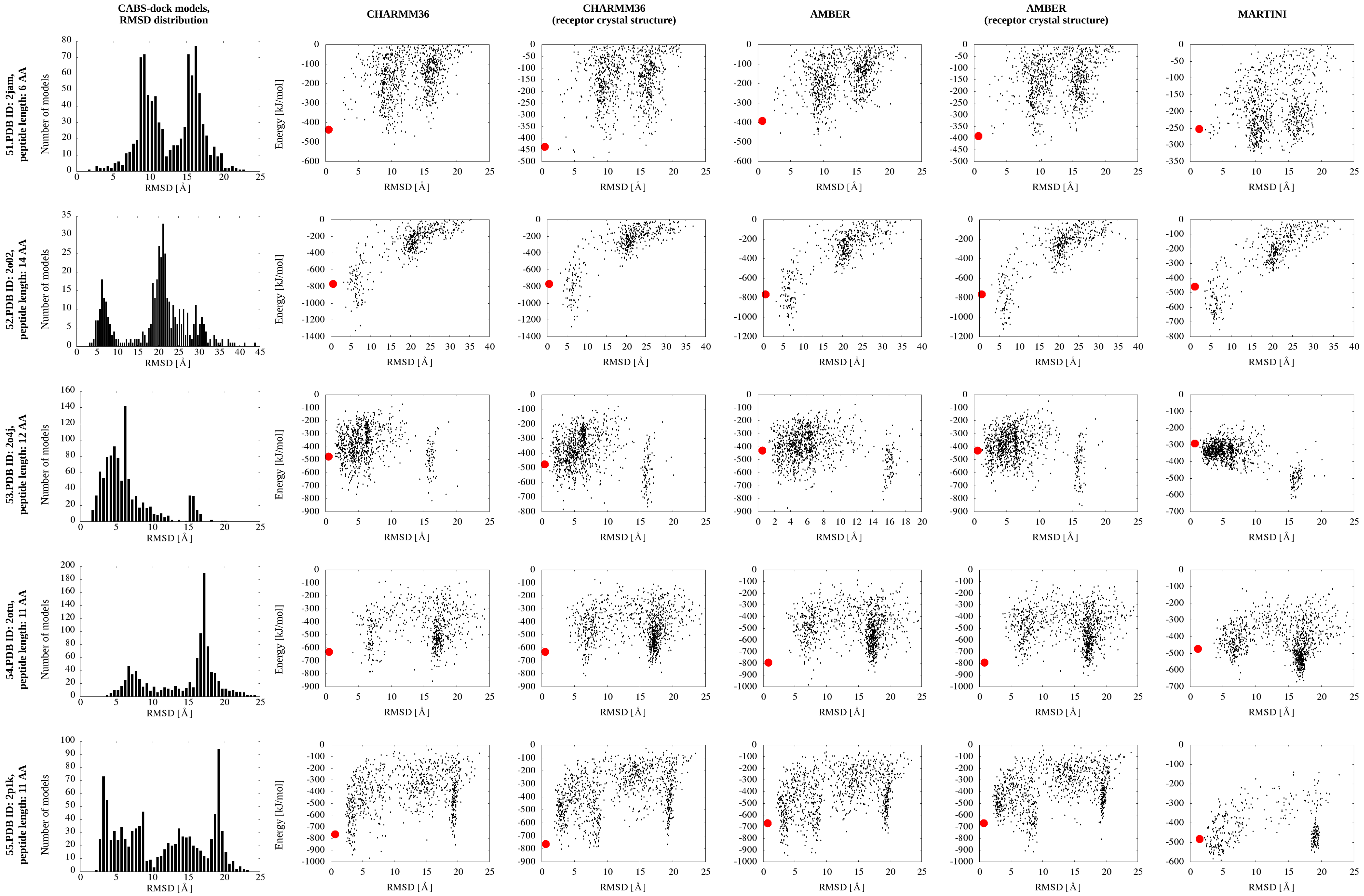

CABS-dock models, RMSD distribution
CHARMM36
CHARMM36 (receptor crystal structure)
AMBER
AMBER (receptor crystal structure)
MARTINI
51.PDB ID: 2jam, peptide length: 6 AA
52.PDB ID: 2oo2, peptide length: 14 AA
53.PDB ID: 2o4j, peptide length: 12 AA
54.PDB ID: 2otu, peptide length: 11 AA
55.PDB ID: 2p1k, peptide length: 11 AA
Number of models
Energy [kJ/mol]
RMSD [Å]

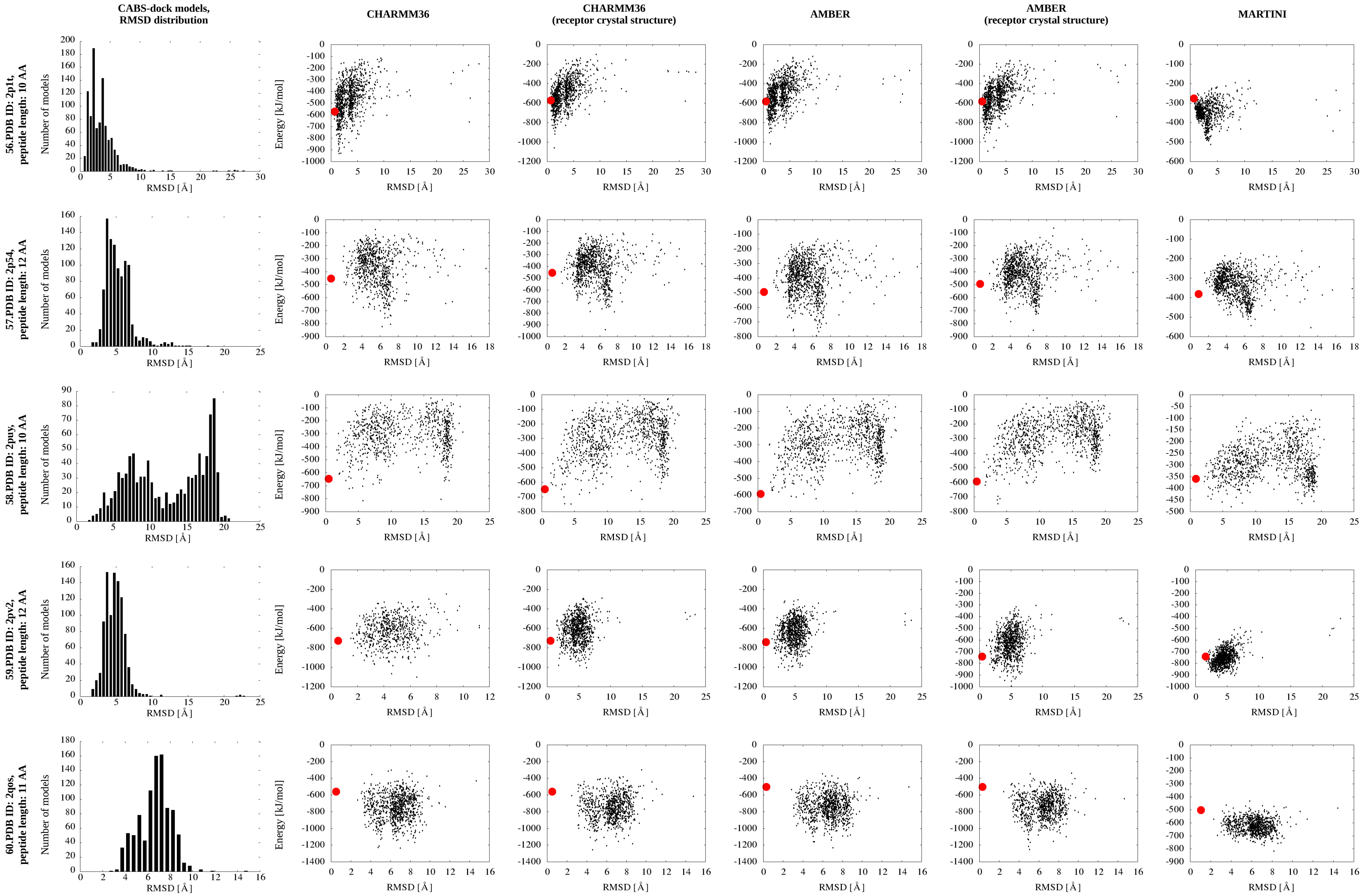

CABS-dock models, RMSD distribution
CHARMM36
CHARMM36 (receptor crystal structure)
AMBER
AMBER (receptor crystal structure)
MARTINI
56.PDB ID: 2p1t, peptide length: 10 AA
57.PDB ID: 2p54, peptide length: 12 AA
58.PDB ID: 2puy, peptide length: 10 AA
59.PDB ID: 2pv2, peptide length: 12 AA
60.PDB ID: 2qos, peptide length: 11 AA
Number of models
Energy [kJ/mol]
RMSD [Å]

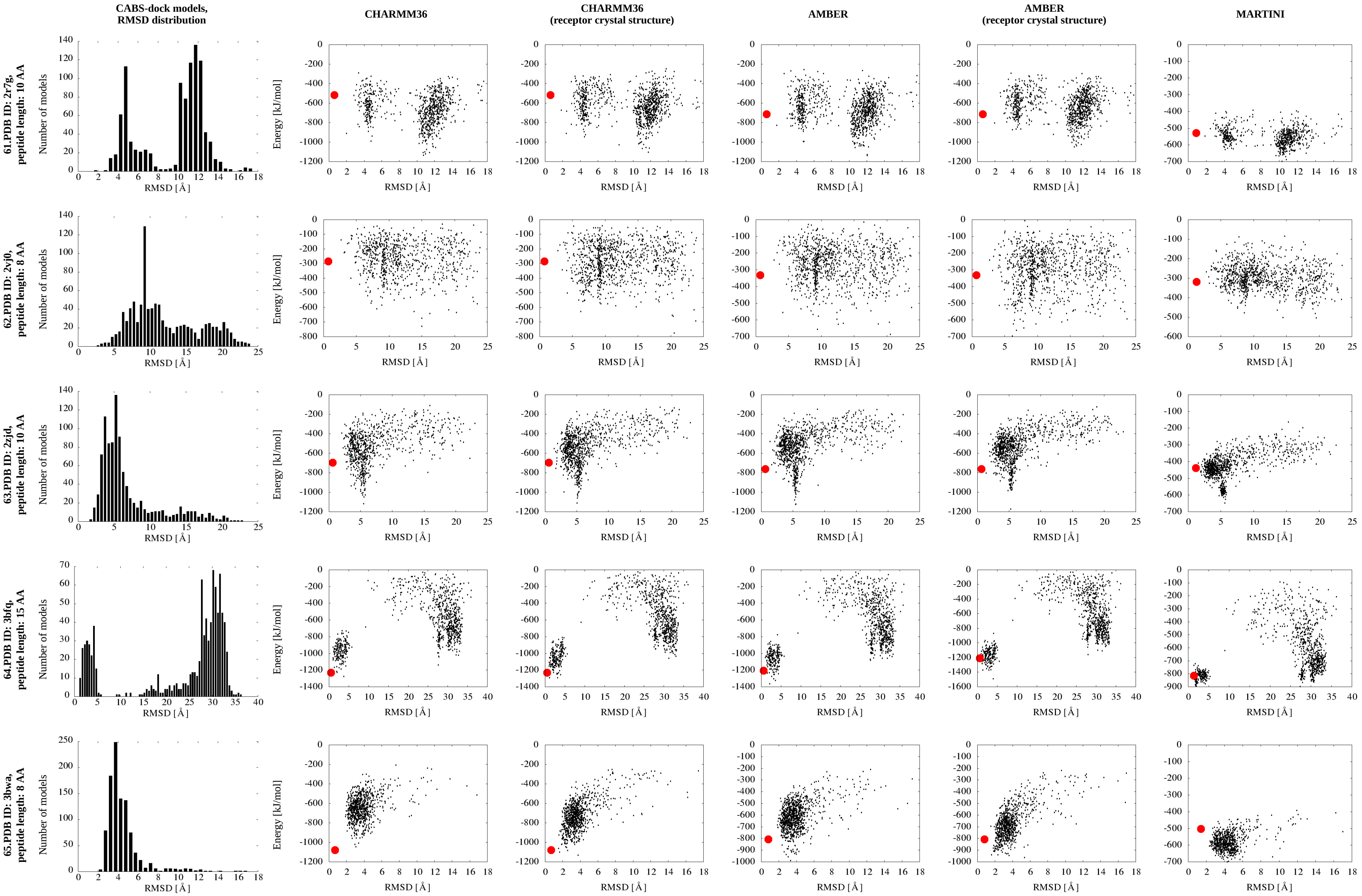

CABS-dock models, RMSD distribution
CHARMM36
CHARMM36 (receptor crystal structure)
AMBER
AMBER (receptor crystal structure)
MARTINI
61.PDB ID: 2r7g, peptide length: 10 AA
62.PDB ID: 2vj0, peptide length: 8 AA
63.PDB ID: 2zjd, peptide length: 10 AA
64.PDB ID: 3bfq, peptide length: 15 AA
65.PDB ID: 3bwa, peptide length: 8 AA
Number of models
Energy [kJ/mol]
RMSD [Å]

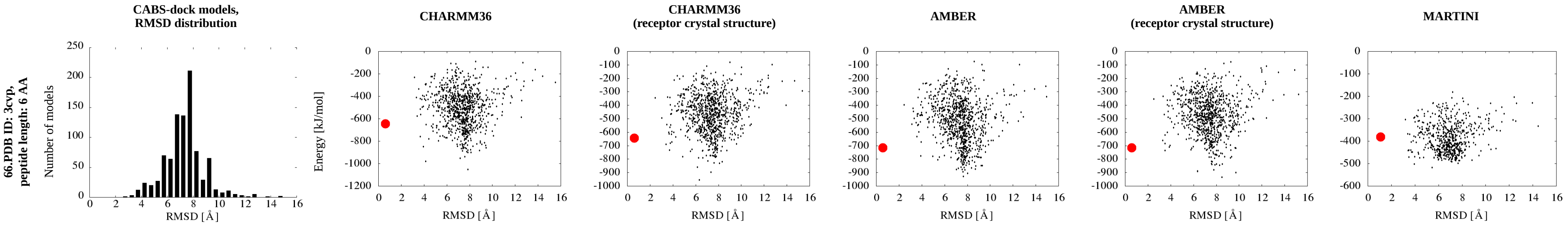
66.PDB ID: 3cvp,
peptide length: 6 AA
CABS-dock models,
RMSD distribution
Number of models
RMSD [Å]
CHARMM36
CHARMM36
(receptor crystal structure)
AMBER
AMBER
(receptor crystal structure)
MARTINI
Energy [kJ/mol]
RMSD [Å]
